# Phloroglucinol Degradation in the Rumen Promotes the Capture of Excess Hydrogen Generated from Methanogenesis Inhibition

**DOI:** 10.3389/fmicb.2017.01871

**Published:** 2017-10-05

**Authors:** Gonzalo Martinez-Fernandez, Stuart E. Denman, Jane Cheung, Christopher S. McSweeney

**Affiliations:** Commonwealth Scientific and Industrial Research Organisation, Agriculture and Food, Queensland Bioscience Precinct, St Lucia, QLD, Australia

**Keywords:** rumen, microbial community, phloroglucinol, CH_4_, H_2_, 16S sequencing

## Abstract

Strategies to manage metabolic hydrogen ([H]) in the rumen should be considered when reducing ruminant methane (CH_4_) emissions. However, little is known about the use of dietary treatments to stimulate rumen microorganisms capable of capturing the [H] available when CH_4_ is inhibited *in vivo*. The effects of the phenolic compound phloroglucinol on CH_4_ production, [H] flows and subsequent responses in rumen fermentation and microbial community composition when methanogenesis is inhibited were investigated in cattle. Eight rumen fistulated Brahman steers were randomly allocated in two groups receiving chloroform as an antimethanogenic compound for 21 days. Following that period one group received chloroform + phloroglucinol for another 16 days, whilst the other group received only chloroform during the same period. The chloroform treatment resulted in a decrease in CH_4_ production and an increase in H_2_ expelled with a shift in rumen fermentation toward higher levels of propionate and formate and lower levels of acetate at day 21 of treatment. Bacterial operational taxonomic units (OTUs) assigned to *Prevotella* were promoted whilst Archaea and Synergistetes OTUs were decreased with the chloroform treatment as expected. The shift toward formate coincided with increases in *Ruminococcus flavefaciens*, *Butyrivibrio fibrisolvens*, and *Methanobrevibacter ruminantium* species. The addition of chloroform + phloroglucinol in the rumen resulted in a decrease of H_2_ expelled (g) per kg of DMI and moles of H_2_ expelled per mol of CH_4_ decreased compared with the chloroform only treated animals. A shift toward acetate and a decrease in formate were observed for the chloroform + phloroglucinol-treated animals at day 37. These changes in the rumen fermentation profile were accompanied by a relative increase of OTUs assigned to *Coprococcus* spp., which could suggest this genus is a significant contributor to the metabolism of this phenolic compound in the rumen. This study demonstrates for the first time *in vivo* that under methanogenesis inhibition, H_2_ gas accumulation can be decreased by redirecting [H] toward alternative sinks through the nutritional stimulation of specific microbial groups. This results in the generation of metabolites of value for the host while also helping to maintain a low H_2_ partial pressure in the methane-inhibited rumen.

## Introduction

Methane is a potent greenhouse gas and also represents a loss of gross feed energy for the ruminant, hence the increasing interest in strategies to manipulate CH_4_ production (Johnson and Johnson, 1995; Gerber et al., 2013). Archaea produce CH_4_ in the rumen mainly by reducing C_1_ compounds with H_2_ (among others substrates) thus maintaining a low hydrogen partial pressure (Janssen, 2010). Although methanogens are the main H_2_ consumers, due to the thermodynamically favorable pathway of CH_4_ formation, there are rumen bacteria, which are also able to use H_2_ as substrate thus generating alternative end products (Leng, 2014). Thus, when CH_4_ production is decreased, [H] might be redirected into alternative sinks nutritionally useful for the ruminant (Ungerfeld, 2015a). However, an excessive increase of the H_2_ partial pressure in the rumen after methane inhibition might have detrimental effects on rumen function (Wolin et al., 1997). Therefore, management of H_2_ accumulation in the rumen is an important strategy to consider when inhibiting CH_4_ emissions from livestock.

A recent published study (Martinez-Fernandez et al., 2016), using chloroform as an antimethanogenic compound, has examined the [H] flow and rumen microbial community responses in cattle fed with hay or supplemented with concentrate. It showed that a CH_4_ reduction of about 30% had no detrimental effects on rumen function in cattle. With both diets a decrease in CH_4_ production was accompanied by an increase in H_2_ expelled. Interestingly, the animals fed with the concentrate diet eructated relatively more H_2_ than the hay diet suggesting inefficient redirection of [H] into other fermentation end products. The main changes observed in rumen fermentation metabolites with both diets were a shift toward propionate and branched-chain fatty acids; an accumulation of formate, particularly with the concentrate diet; and an increase in the concentration of amino acids, organic and nucleic acids. However, although a redirection of [H] was observed, it was predicted that dietary supplements would be needed to increase the capture of excess H_2_ via alternative energy yielding metabolic pathways (Martinez-Fernandez et al., 2016). Failure to capture the excess H_2_ released from the system should be considered as an energy loss to the animal.

Microbial metabolic processes, such as reductive acetogenesis, propionogenesis, reduction of nitrate and sulfate, formate formation, and an increase of microbial biomass production when CH_4_ is inhibited, have been identified as nutritionally useful [H] sinks in the rumen (Newbold et al., 2005; van Zijderveld et al., 2010; Leng, 2014; Gagen et al., 2015; Ungerfeld, 2015b). Another [H] sink which has gained little attention is the reduction of phenolic compounds by rumen microorganisms. Phenolic compounds, such as flavonoids, are present in many of the forage plants consumed by ruminants. Rumen bacteria, in some cases are capable of transforming these compounds into energy yielding products for the host (Murdiati et al., 1992; McSweeney et al., 2001). Flavonoids undergoing microbial degradation in the rumen generally form phloroglucinol as an intermediate metabolite (Supplementary Figure 1). Several studies have shown that specific rumen bacteria are able to reduce phloroglucinol using H_2_ or formate as electron donors thus yielding acetate as the terminal product (Tsai and Jones, 1975; Patel et al., 1981; Krumholz and Bryant, 1986). However, there is scarce information about the use of unconventional dietary treatments that may stimulate rumen microorganisms capable of capturing the [H] available when CH_4_ is inhibited *in vivo*.

The aims of the present study were to analyze the effect of phloroglucinol on CH_4_ production, [H] flows and subsequent responses in rumen fermentation and microbial community composition in cattle when methanogenesis is inhibited. It was hypothesized that less H_2_ would be expelled by the chloroform + phloroglucinol-treated animals due to a shift in fermentation toward acetate by the reduction of the phenolic compound.

## Materials and Methods

The experimental protocol complied with the Australian Code for the Care and Use of Animals for Scientific Purposes (eighth edition, 2013) and was approved by the local Animal Experimentation and Ethics Committee (A08/2014).

### Treatments

The antimethanogen compound used was a halogenated hydrocarbon (chloroform) entrapped in a β-cyclodextrin matrix (6–7% w/w chloroform) as described by Martinez-Fernandez et al. (2016). The phenolic compound used was phloroglucinol (Thermo Fisher Scientific, Scoresby, VIC, Australia; purity of 99%).

### Experimental Design and Sampling

Eight rumen fistulated Brahman (*Bos indicus*) steers (live weight, 365 ± 12 kg) at Lansdown Research Station (Townsville, QLD, Australia) were randomly allocated in two groups (four animals per group), receiving a diet *ad libitum* with a ratio 60:40 forage:concentrate (Rhodes grass hay; chemical composition: dry matter (DM), 917 g/kg fresh matter; in g/kg of DM: organic matter, 814; crude protein (CP), 109; neutral detergent fiber (NDF), 651; acid detergent fiber (ADF), 351. Concentrate; Ridley AgriProducts Pty Ltd., Brisbane, QLD, Australia. Ingredients (g/kg): barley (458), sorghum (200), molasses (30), wheat (200), legume hulls (80), urea (2.5); concentrate chemical composition: DM, 887 g/kg fresh matter; in g/kg of DM: CP, 114; NDF, 199; ADF, 130; and fat, 24). Animals were adapted to the diet over 30 days and then placed into individual pens for the measurement of individual intakes (10 days) and treated through the cannula with cyclodextrin (2 g/100 kg LW). On days 9 and 10, animals were placed into open-circuit respiration chambers for measurement of CH_4_ and H_2_ production and collection of rumen samples. Following the initial control period animals received the chloroform through the rumen-cannula, increasing the dose progressively during 21 days (treatment period 1, 1.6 g/100 kg LW, divided into two doses per day) with the last 2 days confined in open-circuit respiration chambers for direct measurement of CH_4_ and H_2_ production and rumen fluid collection. After that period, one group received the chloroform + phloroglucinol for 16 days, whilst the other group received only the chloroform treatment (treatment period 2). Phloroglucinol was placed in the rumen through the cannula twice per day, progressively increasing the dose during the first 10 days up to 75 g/100 kg LW (estimated rumen concentration 40 mM). The last 2 days of that period rumen samples and CH_4_ and H_2_ measurements were taken as previously described.

Rumen fluid samples (approximately 60 ml per animal) were collected through the cannula of the animal using a probe (covered with two layers of cheesecloth) at 3 h post feeding during confinement in respiration chambers to determine the effect on rumen fermentation parameters and rumen microbial communities. Rumen samples were stored at -20°C for short chain fatty acid (SCFA) and NH_3_-N analyses. Additionally, 20 ml were kept at -80°C prior to DNA extractions.

Four open circuit respiration chambers were used to determine CH_4_ and H_2_ production from individual steers as described by Martinez-Fernandez et al. (2016).

### Chemical Analysis

The feed samples were dried in a forced-air oven at 65°C prior to grinding. Feed samples were ground through a 1-mm sieve before analysis. DM, ash, NDF, ADF, fat, and total nitrogen contents were analyzed by Symbio Alliance (Eight Mile Plains, QLD, Australia) following the accredited methods CF006.1, CF007, CF038.1, CF038.3, CF004, and CF003.2, respectively [AOAC International (2005) official methods: 925.10, 923.03, 920.39, 990.03, 2002.04, and 973.18]. The nitrogen values were converted to CP by a multiplication factor of 6.25.

Rumen fluid concentrations of SCFAs (acetate, propionate, *n*-butyrate, isobutyrate, isovalerate, and *n*-valerate) were measured by gas chromatography (GC) as described by Gagen et al. (2014). Isovalerate (3-methyl butyrate) includes 2-methyl butyrate, which co-elutes. The rumen NH_3_-N concentration was determined by the colorimetric method of Chaney and Marbach (1962).

An UltiMate^®^3000 HPLC system (Dionex, Sunnyvale, CA, United States) with a dedicated Photodiode Array Detector and an Autosampler was used to determine the concentration of formic acid in rumen fluid as described by Gagen et al. (2014).

### Calculation of [H] Redirection and Non-carboxyl SCFA Carbons

The differences between treatments regarding incorporation of [H] into SCFA (HUSr) and formate (HUFr) were estimated using the concentrations of metabolites as a proxy since actual production rates were not measured. The stoichiometry was calculated as described by Martinez-Fernandez et al. (2016). The CH_4_ gas production (GP; mol/day) and H_2_ gas production (GP; mol/day) was used to calculate the ratio between [H] redirected to H_2_ expelled/CH_4_ decreased (Martinez-Fernandez et al., 2016).

### DNA Extractions

DNA extractions from rumen samples were performed using the cetyltrimethylammonium bromide (CTAB) method of Brookman and Nicholson (2005) with minor modifications as follows: samples were centrifuged (13,000 × *g* for 5 min), and the supernatant was removed before DNA extraction. Cells were homogenized with 200 mg of silica-zirconium beads (1:1 mixture of 0.1- and 1.0-mm beads; Biospec, Bartlesville, OK, United States) and 800 μl of CTAB buffer in a Mini-Beadbeater-8 (Biospec) on maximum speed for 2 min, twice. Samples were incubated at 70°C for 20 min and centrifuged at 10,000 × *g* for 10 min, and the supernatant was mixed with 500 μl of 25:24:1 phenol:chloroform:isoamyl alcohol (Fluka BioChemika, Buchs, Switzerland).

### 16S rDNA and Statistical Analyses

High throughput sequencing platforms, barcoding procedures for sample recognition and phylogenetic analysis of the 16S rDNA gene were used to characterize the microbial populations present in the rumen for the control and treatment periods. The V4 region of the 16S rRNA gene was targeted using specific primers (Kozich et al., 2013). Each individual DNA sample was amplified using the specific primers and a unique barcode combination. Afterward, amplification products were visualized by performing gel electrophoresis. Product quantities were calculated and an equal molar amount of each product was pooled. The pooled products were run in a 1% agarose gel and bands were visualized and excised under blue light transillumination. The amplicons were gel purified with QIAquick Gel extraction Kit (Qiagen, Hilden, Germany) prior to submission for Illumina Miseq (Macrogen Inc., South Korea).

Paired end short read sequence data generated on the Illumina Miseq was processed using the USEARCH package (Edgar, 2010). De-multiplexed paired end sequences were first merged prior to sequence quality filtering, followed by denoising (error correction) and chimera checking and clustering of sequences to operational taxonomic units (OTUs) of 97% similarity. Taxonomic assignment of sequences was performed against the Greengenes database (McDonald et al., 2012). Additional analysis of OTUs was performed in the R packages vegan, Phyloseq and DESeq2 and the ggplot2 graphics package (McMurdie and Holmes, 2013; Oksanen et al., 2013; Love et al., 2014; Wickham, 2016). The significances of grouping in the PCoA plots were tested by analysis of dissimilarity (ADONIS) with 999 permutations. The sequences obtained in this paper have been deposited in the European Nucleotide Archive under the accession number (PRJEB20458).

The effect of treatment was analyzed for CH_4_/H_2_ production, DMI, ADWG, and rumen fermentation metabolites. Chloroform only and chloroform + phloroglucinol groups were compared as a univariate model using the GLM procedure of SPSS (IBM, version 21.0), the treatment was considered the fixed effect with the animal as experimental unit. Data from the chloroform only treatment at day 21 and their respective controls (non-treated period) were analyzed separately as a repeated-measures analysis using the linear mixed-model of SPSS (IBM, version 21.0), with the animal as the experimental unit. Effects were declared significant at *P* < 0.05 and *P*-values between 0.05 and 0.10 were considered as a trend.

## Results

### Ruminal Fermentation and Gas Production

No significant effects were observed on DMI, average daily weight gain, CH_4_ and H_2_ production (g/kg DMI), and fermentation parameters between experimental groups at either the control period or at day 21 of chloroform treatment before supplementation with phloroglucinol (Supplementary Tables 1, 2). A significant decrease (*P* ≤ 0.05) in CH_4_ production (g/kg DMI) (40% reduction) and increase in H_2_ (1.2 H_2_ g/kg DMI) were observed in both groups of animals treated with chloroform compared with their respective control period. The rumen fermentations parameters showed an increase (*P* ≤ 0.05) of propionate, branched-chain fatty acids, and formate in chloroform-treated animals compared with the control period (Supplementary Table 3).

Animals that continued to receive only chloroform continued to maintain lower CH_4_ emissions, but changes were observed in VFA profiles between days 21 and 37. With continued chloroform treatment there was a decrease in the molar percentage of propionate with concurrent increases in acetate and formate concentration (**Table 1** and Supplementary Table 3). Conversely those animals supplemented with chloroform + phloroglucinol at day 37 had a significant (*P* ≤ 0.05) reduction in both the amount of H_2_ expelled (g) per kg of DMI and moles of H_2_ expelled per mol of CH_4_ decreased compared with the chloroform only treated animals (**Table 1**). Rumen fermentation parameters showed a significant increase in acetate and reduction in formate concentration (*P* ≤ 0.05) in animals treated with chloroform + phloroglucinol compared with the chloroform only group. Daily weight gain increased significantly (*P* ≤ 0.05) in chloroform + phloroglucinol treated animals compared with the chloroform only treated group (1.38 vs 0.438 kg/day, respectively) at day 37.

**Table 1:** Chloroform and chloroform + phloroglucinol effects on DMI, CH_4_, H_2_, daily weight gain, and rumen fermentation parameters in animals at day 37.

	Chloroform + phloroglucinol	Chloroform	SEM	*P*-value
*N* (number of animals)	4	4		
DMI, kg	8.2	7.3	0.35	0.23
CH_4_ (g/kg DMI)	11.8	13.8	0.61	0.11
H_2_ (g/kg DMI)	0.44	0.89	0.116	0.042
mol H_2_/mol CH_4_ decreased	0.09	0.21	0.012	0.004
Daily weight gain, kg	1.38	0.453	0.211	0.012
Formate (mM)	0.83	11.3	2.76	0.046
pH	6.43	6.58	0.06	0.27
NH_3_-N (mg/100 ml)	14.8	12.9	1.08	0.43
Total SCFA (mM)	94.9	90.4	4.32	0.63
**SCFA (mol/100 mol)**				
Acetate	65.2	60.7	1.03	0.012
Propionate	19.4	22.8	1.37	0.24
i-Butyrate	0.7	1.1	0.212	0.39
Butyrate	11.6	10.7	0.77	0.59
i-Valerate	0.9	1.2	0.282	0.56
Valerate	1.43	1.60	0.293	0.80
Caprionate	0.88	1.42	0.344	0.48
A:P	3.46	2.74	0.268	0.19


**FIGURE 1:**
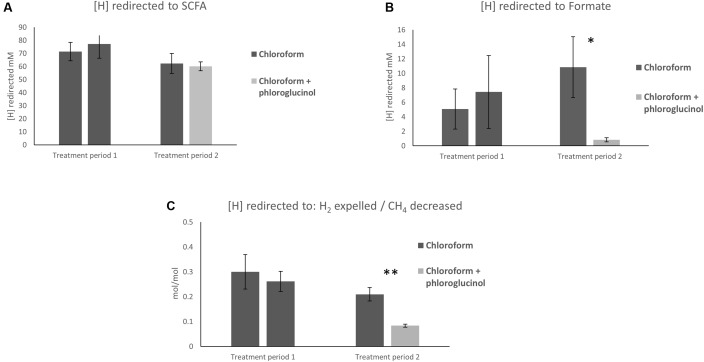
Effect of treatments on **(A)** redirection of [H] to SCFA, **(B)** formate, and **(C)** the ratio between [H] redirected to H_2_ expelled/CH_4_ decreased. Asterisks “^∗^” and “^∗∗^” denote significant differences between treatments, *P* < 0.05 and *P* < 0.010, respectively.

The calculations of [H] flows showed no significant differences in the [H] redirection into SCFA between chloroform + phloroglucinol treated animals and the chloroform only group (**Figure 1A**). Whereas, the chloroform + phloroglucinol treated animals showed a significant (*P* ≤ 0.05) decrease in [H] redirected into formate compared to the chloroform only treatment (**Figure 1B**). Furthermore, a significant decrease (*P* ≤ 0.01) of [H] redirected into H_2_ per mol of CH_4_ decreased (**Figure 1C**) was observed for the chloroform + phloroglucinol group compared with the chloroform only treated group.

### Microbial Community

The diversity analysis of the rumen microbiota showed that total microbial species richness and evenness (Shannon and Simpson, respectively) were not impacted significantly either by treatment or time (Supplementary Figure 2).

The structure of the microbiome as determined by non-phylogenetic beta diversity analysis (Bray–Curtis) showed the largest variance was due to time (treatment effect), with the control period more different than treatments at 21 and 37 days (Supplementary Figure 3). Time explained 14% of the variance from the centroid (*P* < 0.001), while the animal groups only explained 4% (*P* = 0.03), showing a separation between chloroform and chloroform + phloroglucinol treated animals at day 37 (beta dispersions; *P* = 0.151).

**FIGURE 2:**
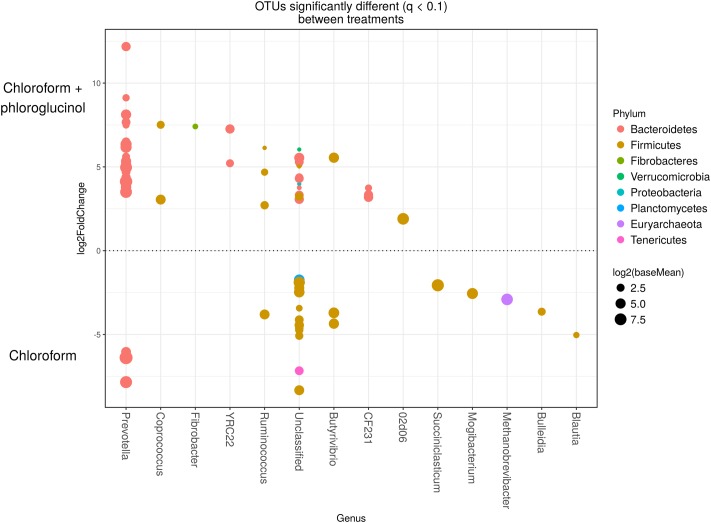
OTUs significantly different (*q* < 0.1 FDR) between chloroform + phloroglucinol and chloroform treated-animals. Upper axis represents OTUs with a log2 fold positive difference for chloroform + phloroglucinol treatment relative to chloroform while the lower *y*-axis is the negative fold difference of the chloroform + phloroglucinol relative to chloroform. Each point represents a single OTU colored by phylum and grouped on the *x*-axis by taxonomic genus level, size of point reflects the log2 mean abundance of the sequence data.

**FIGURE 3:**
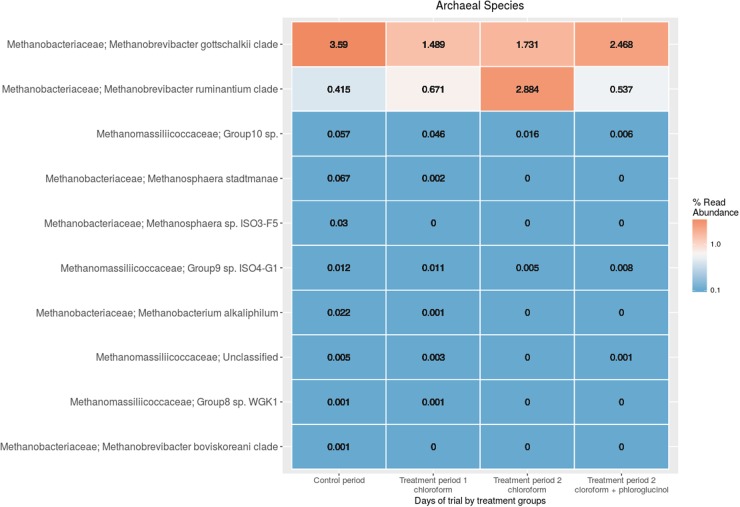
Rumen methanogen community heat maps of the most abundant OTUs using regularized log transformed values from DeSeq2 at control period, treatment period 1 and treatment period 2.

The comparison of the rumen microbiome showed a shift in specific OTUs between the chloroform treated animals and the control period, with an increase of OTUs classified in the *Prevotella* genus and a decrease in OTUs assigned to the Archaea domain in the chloroform animals compared with the control period (Supplementary Figure 4). Animals that continued with the chloroform only treatment to day 37 showed decreases in OTUs assigned to *Prevotella* and increases for those assigned with *Ruminococcus*, *Butyrivibrio*, and *Methanobrevibacter* (Supplementary Figures 5–7). The chloroform + phloroglucinol treatment at day 37 was associated with an increase in OTUs affiliated with the genera *Prevotella*, *Ruminococcus*, *Fibrobacter*, CF231, YRC22, and *Coprococcus* compared to animals receiving only chloroform, with OTUs assigned to *Coprococcus* and *Prevotella* showing the greatest fold change (**Figure 2**). Compared to chloroform only treatment, the addition of phloroglucinol altered the methanogen rank abundance back to one more representative of the control animals (**Figure 3**). Within the *Methanobrevibacter* genus, *Methanobrevibacter gottschalkii* and Methanomassiliicoccaceae associated OTUs remained suppressed, while *Methanobrevibacter ruminantium* OTUs were significantly increased on day 37 (**Figure 3**).

**FIGURE 4:**
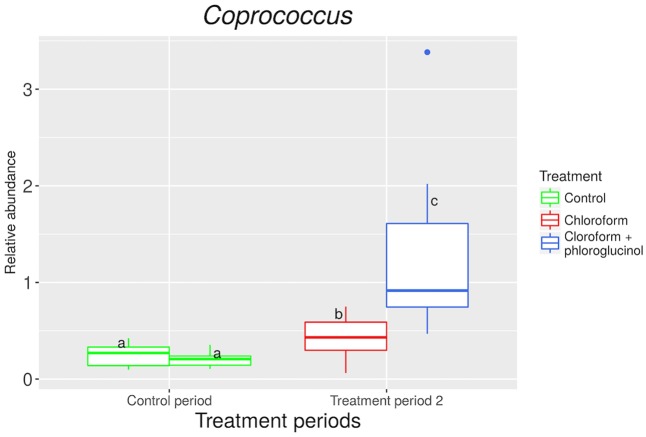
Relative abundance of OTUs assigned to *Coprococcus* genus at control period (untreated animals) and treatment period 2 (chloroform + phloroglucinol and chloroform-treated animals). ^a,b^Letters denote significant differences between groups, bars that do not share the same letter are significantly different from each other (*P* < 0.05).

The relative abundance of the *Coprococcus* genus was significantly (*P* < 0.001) increased in the chloroform + phloroglucinol treated animals at day 37 compared with the chloroform-only treatment at day 37 and the control period (**Figure 4**). A significant (*P* < 0.001) decrease in the Synergistetes phylum was observed with chloroform and chloroform + phloroglucinol treated animals at day 37 compared with the untreated animals at day 0 (Supplementary Figure 8). Synergistetes members were also more abundant (*P* = 0.003) in chloroform + phloroglucinol treated animals compared with the chloroform only group at day 37.

## Discussion

Inhibition of methanogenesis in ruminants using halogenated compounds has previously shown a redirection of [H] toward propionate and branched-chain fatty acids with increased H_2_ eructation from the rumen (Mitsumori et al., 2012; Martinez-Fernandez et al., 2016). Likewise, the administration of chloroform to animals in this trial produced a similar response, thus providing a model system for testing the ability to alter rumen metabolic pathways through the capture of excess H_2_. A number of alternative [H] sinks have been identified for when methanogenesis is inhibited in ruminant livestock such as: acetogenesis (Joblin, 1999; Fonty et al., 2007; Gagen et al., 2015), propionogenesis (Newbold et al., 2005), nitrate reduction to ammonia (Morvan et al., 1996; Anderson et al., 2003; van Zijderveld et al., 2010; Latham et al., 2016), sulfate reduction to hydrogen sulfite (van Zijderveld et al., 2010), formate formation (Leng, 2014), and increases of microbial biomass production (Ungerfeld, 2015b). However, there is scarce information about the amount of [H] redirected to these metabolic processes or emitted as H_2_ gas under *in vivo* conditions. While the rumen microbiota can adapt to inhibition of CH_4_ formation by redirecting a proportion of [H] into energy-yielding metabolites, an important proportion of the unutilized [H] accumulates and is eructated as H_2_ gas, which still represents an energy loss to the animal. This study demonstrates for the first time *in vivo* that under methanogenesis inhibition, H_2_ gas accumulation can be reduced by redirecting [H] toward alternative sinks through the nutritional stimulation of microbial groups that generate metabolites of value to the host. Therefore, the use of dietary treatments to promote key rumen microbial groups, may be a practical way of capturing [H] that would normally be lost as H_2_ gas when a strategy of directly inhibiting methanogens in the rumen is employed to reduce CH_4_ formation.

Interestingly, an increase in daily weight gain was observed for the chloroform + phloroglucinol treated animals, indicating that the [H] redirection toward an alternative sink facilitated the utilization of a substrate, which is not normally regarded as an energy-yielding nutrient for the host. Although the increase in weight gain is a promising result, it should be interpreted with caution due to the relatively short length of the trial and small number of animals used. A recent trial with dairy cattle using 3-nitrooxypropanol to reduce CH_4_ emissions produced an 80% increase on the average body weight gain over the experimental period (Hristov et al., 2015). While this most likely indicates a shift in metabolism to end products of greater nutritional or metabolic value, the authors also observed a 64-fold increase in hydrogen emissions, suggesting that further efficiencies could be gained. However, to confirm this hypothesis further *in vivo* research on rumen digestibility and metabolism should be carried out.

In relation to the redirection of [H], the inclusion in the rumen of the phenolic substrate phloroglucinol resulted in greater acetate formation with less H_2_ expelled per mole of CH_4_ decreased, and decreased formate concentration in the rumen. This is in agreement with *in vitro* studies (Patel et al., 1981; Krumholz and Bryant, 1986) that have shown that rumen microorganisms were able to metabolize phloroglucinol to acetate by consuming H_2_ or formate as reducing agents. It should be noted that the formula for calculation of the [H] used in SCFA formation (Martinez-Fernandez et al., 2016) does not account for the acetate produced through reductive processes, such as acetogenesis or reduction of phenolic compounds, which most likely explains why there was no difference in the calculated flow of [H] into these organic acids between the treatments. Chloroform + phloroglucinol treated animals expelled per day 1.42 mol of H_2_ less than the chloroform only treated group, which is lower than the predicted moles of H_2_ (2.18) used to degrade the amount of phloroglucinol provided per day (1 molecule phloroglucinol + 1 molecule H_2_ = 2 molecules acetate + 2 molecules carbon dioxide (Tsai et al., 1976; Conradt et al., 2016) (Supplementary Figure 1). This discrepancy might indicate that phloroglucinol was not totally metabolized, however, near complete degradation of similar quantities of phenolic compounds have been reported previously (Murdiati et al., 1992). It is more likely that both formate and H_2_ were used to reduce phloroglucinol based on the lower formate concentrations in the chloroform + phloroglucinol treated animals (0.83 vs 11.3 mM) compared with chloroform only. Formate is produced during normal rumen fermentation and is mainly consumed by methanogens to produce CH_4_ (Hungate et al., 1970; Asanuma et al., 1998). Similar to the current study, an increase in formate has been reported previously in steers which were treated with chloroform alone to reduce methanogenesis, particularly with concentrate-supplemented diets (Martinez-Fernandez et al., 2016). Our results support the hypothesis that increased formate accumulation represents a response by the rumen microbiota to control H_2_ partial pressures in the rumen, by acting as a hydrogen sink (Leng, 2014; Ungerfeld, 2015b; Martinez-Fernandez et al., 2016).

Members of the Synergistetes phylum are sensitive to H_2_ partial pressures within the rumen and generally are reduced when the H_2_ partial pressure increases or methanogenesis is restricted (Denman et al., 2015; Wallace et al., 2015; Martinez-Fernandez et al., 2016). Similar reductions in the Synergistetes phylum were observed in this study with the chloroform-only treatment and increased H_2_ partial pressures. The addition of phloroglucinol and redirection of [H] led to lower partial pressures of H_2_ and probably resulted in increases in the Synergistetes phylum further supporting their significance as a key indicator group around hydrogen transactions.

Of particular interest, was the greater increase in formate concentration when the animals were exposed to longer periods of chloroform, allowing for subsequent increases in the acetate:propionate ratio, presumably driven by the decrease in H_2_ partial pressures. Fibrolytic *Ruminococcus* species are able to alter their fermentative pathways to provide alternative reducing equivalent sinks, including the production of formate and ethanol to relieve the inhibition of the reoxidation of NADH (Miller and Wolin, 1974; Shi et al., 1997). With the higher partial pressures of H_2_ observed in the rumen for the chloroform treated animals, the shift to increased formate might be linked with increases of *Ruminococcus flavefaciens*, *Butyrivibrio fibrisolvens*, and *M. ruminantium* species. Methanogen species capable of utilizing formate are also able to produce formate from CO_2_ and H_2_ under conditions of high H_2_ partial pressures and when inhibited with halogenated hydrocarbons (Thiele and Zeikus, 1988; Bleicher and Winter, 1994). The observed increase in the relative abundance of *M. ruminantium* over the predominant rumen methanogen *M. gottschalkii*, which is incapable of formate utilization, likely reflects the activation of this mechanism. By converting H_2_ and CO_2_ to formate while H_2_ is in excess and methanogenesis is impeded may ensure survival until conditions become more favorable for producing CH_4_, thus providing *M. ruminantium* with a competitive advantage over non-formate utilizing methanogens. The addition of phloroglucinol altered these conditions by redirecting [H] away from formate and reducing the H_2_ partial pressure which allowed *M. gottschalkii* to outcompete *M. ruminantium* and once again become the dominant methanogen species in this system. The changes in the microbiota illustrate the ability of the rumen to approach a new equilibrium by adapting to the altered environmental conditions. Of course, this also means that these changes might not correspond to a fully adapted and stable rumen microbiome as is illustrated by the continued shift in the microbiome for the chloroform only group between days 21 and 37. Longer trial periods would need to be studied to fully answer if the rumen has reached a stable microbiota.

Phloroglucinol is a phenolic compound, formed through the degradation of complex plant molecules such as flavonoids, which are commonly present in the diet of the grazing ruminant (Tsai et al., 1976). It is also present at high levels in brown algae (*Ecklonia cava*) which is widely harvested for its medicinal effects (Park et al., 2012). In the rumen, microorganisms degrade this compound predominately to produce acetate and CO_2_ as end-products. Our findings are in accordance with previous *in vitro* studies (Tsai et al., 1976; Patel et al., 1981; Krumholz and Bryant, 1986), which observed this increase in acetic acid concentration when phloroglucinol was metabolized by rumen microorganisms. Several rumen bacteria have been identified as phloroglucinol degraders and were classified as *Eubacterium oxidoreducens*, *Streptococcus bovis*, and *Coprococcus* spp. (Tsai et al., 1976; Patel et al., 1981; Krumholz and Bryant, 1986). Krumholz and Bryant (1986) reported that *E. oxidoreducens* required H_2_ or formate to degrade phloroglucinol to acetate. The analysis of the rumen microbiome in this study did not identify *E. oxidoreducens* but did reveal several OTUs assigned to *Coprococcus* spp. increased within the chloroform + phloroglucinol treated animals, suggesting the genus is a significant contributor to the metabolism of this phenolic compound. Tsai et al. (1976) found that *Coprococcus* spp. were able to degrade 1 molecule of phloroglucinol to 2 molecules of acetic acid and 2 molecules of carbon dioxide. A more detailed study by Patel et al. (1981) showed that phloroglucinol is initially reduced to dihydrophloroglucinol by *Coprococcus* spp. using NADPH as the electron donor. This would help alleviate the increase in NADPH in the rumen generated by the inhibition of methanogenesis through its oxidation to NADP^+^ (Chalupa, 1977). Furthermore, a recently published study (Conradt et al., 2016) identified three phloroglucinol reductases belonging to the family of NADPH dehydrogenases/reductases involved in the anaerobic degradation of phloroglucinol which involves hydrolytic ring cleavage to 3-hydroxy-5-oxohexanoic and then acetate formation. However, to confirm our hypothesis, further studies using metagenomics and transcriptomic approaches should be carried out to evaluate the functional genes in *Coprococcus* spp. associated with the NADPH-dependent reduction of phloroglucinol and the redirection of H_2_ in the rumen. Furthermore, as the limitations of the trial did not allow for the inclusion of a phloroglucinol only treatment, we cannot ascertain if these effects may have also been possible in the presence of functional methanogenesis and lower H_2_ partial pressures. Indeed, a calculated Δ*G*° of -158 kJ/mol phloroglucinol to acetate indicates this reaction is likely to be very favorable thermodynamically and may even occur in the presence of hydrogenotrophic methane formation (Kaiser and Hanselmann, 1982; Krumholz and Bryant, 1986).

The *Coprococcus* genus is also involved in other important metabolic pathways in the rumen. Recently, Shabat et al. (2016) found a greater proportion of *Coprococcus* species (*Coprococcus catus*) in the rumen of dairy cows with lower CH_4_ emissions and higher feed efficiencies. In the more feed efficient cows there were an increase in abundance of genes aligned to the acrylate pathway, which involves the conversion of lactate to propionate, and were annotated as *C. catus*. In general, the acrylate pathway rather than the succinate pathway for propionate production was more dominant in the efficient animals (Shabat et al., 2016). Other metabolic pathways in which *Coprococcus* spp. are also involved, is the degradation of nitrotoxins in the rumen (Majak and Cheng, 1981) through reductive processes. These and our findings suggest that *Coprococcus* genus is involved in key metabolic pathways in the rumen, and they should be considered when developing strategies to promote microorganisms which might improve the rumen efficiency and metabolize plant toxins in ruminants.

## Conclusion

The present study in cattle demonstrated that under anti-methanogenesis conditions the addition of phloroglucinol into the rumen can stimulate microbial groups which utilize H_2_ and formate as reductants in the metabolism of phloroglucinol to acetate and thus decrease the partial pressure of H_2_ in the rumen and the amount of this gas eructated by the animal. This demonstrates that the negative impact of H_2_ accumulation in the rumen under suppression of methanogenesis can be ameliorated by the provision of novel compounds that have nutritional value for the animal when degraded by reductive processes.

## Author Contributions

CM, SD, and GM-F conceived and designed the experiments and analytical approaches. GM-F performed the animal trial. GM-F and JC analyzed the biological samples. SD and GM-F analyzed the data. GM-F, CM, and SD wrote the manuscript. All authors agree to be accountable for all aspects of the work.

## Conflict of Interest Statement

The authors declare that the research was conducted in the absence of any commercial or financial relationships that could be construed as a potential conflict of interest.
