# Supplementary material for: Phloroglucinol Degradation in the Rumen Promotes the Capture of Excess Hydrogen Generated from Methanogenesis Inhibition
Source: Front Microbiol. 2017 Oct 5;8:1871. doi: 10.3389/fmicb.2017.01871 (PMC5633678; doi:10.3389/fmicb.2017.01871)
Supplement: Supplementary file 1 [file Data_Sheet_1.docx]

***Supplementary Material***

**Phloroglucinol degradation in the rumen promotes the capture of excess hydrogen generated from methanogenesis inhibition**

**Gonzalo Martinez-Fernandez^*^, Stuart E. Denman, Jane Cheung, and Christopher S. McSweeney**

CSIRO, Agriculture and Food, Queensland Bioscience Precinct, St Lucia, QLD, Australia

*** Correspondence:** Gonzalo Martinez Fernandez, CSIRO, Agriculture & Food, Queensland Bioscience Precinct, 306 Carmody Road, St Lucia, QLD, 4067, Australia. Email:gonzalo.martinezfernandez@csiro.au

1. **Supplementary Tables and Figures**
   1. **Supplementary Tables**

**Supplementary Table 1.** Group effects on DMI, CH_4_, H_2_, average daily weight gain (ADWG) and rumen fermentation parameters in steers at control period.

|  | Control | |  |  |
| --- | --- | --- | --- | --- |
|  | Group 1 | Group 2 | SEM | P-value |
| *N* (number of animals) | 4 | 4 |  |  |
| DMI, kg | 7.5 | 7.1 | 0.30 | 0.50 |
| CH_4_ (g/kg DMI) | 22.2 | 22.3 | 0.92 | 0.94 |
| H_2_ (g/kg DMI) | 0.13 | 0.17 | 0.019 | 0.33 |
| ADWG, kg | 1.00 | 0.917 | 0.150 | 0.72 |
| Formate (mM) | 0.00 | 0.00 | 0.0 | 0.99 |
| pH | 6.59 | 6.37 | 0.10 | 0.33 |
| NH_3_-N (mg/100 mL) | 9.6 | 11.7 | 1.32 | 0.46 |
| Total SCFA (mM) | 83.9 | 99.8 | 4.83 | 0.10 |
| SCFA (mol/100 mol) |  |  |  |  |
| Acetate | 62.9 | 68.3 | 1.85 | 0.16 |
| Propionate | 22.2 | 20.4 | 1.53 | 0.59 |
| i-Butyrate | 0.96 | 0.80 | 0.066 | 0.24 |
| Butyrate | 11.55 | 8.37 | 0.89 | 0.06 |
| i-Valerate | 0.99 | 0.71 | 0.076 | 0.06 |
| Valerate | 0.96 | 0.98 | 0.062 | 0.87 |
| Caprionate | 0.40 | 0.49 | 0.106 | 0.71 |
| A:P | 2.95 | 3.53 | 0.33 | 0.41 |

Group 1: Treated with chloroform + phloroglucinol from day 22.

Group 2: Treated with chloroform from day 22.

**Supplementary Table 2.** Group effects on DMI, CH_4_, H_2_, average daily weight gain (ADWG) and rumen fermentation in animals treated with chloroform at day 21.

|  | Chloroform | |  |  |
| --- | --- | --- | --- | --- |
|  | Group 1 | Group 2 | SEM | P-value |
| *N* (number of animals) | 4 | 4 |  |  |
| DMI, kg | 6.8 | 6.8 | 0.31 | 0.95 |
| CH_4_ (g/kg DMI) | 13.8 | 12.6 | 1.04 | 0.61 |
| H_2_ (g/kg DMI) | 1.15 | 1.23 | 0.127 | 0.77 |
| mol H_2_/ mol CH_4_ decreased | 0.26 | 0.30 | 0.038 | 0.65 |
| ADWG, kg | 0.988 | 1.18 | 0.158 | 0.58 |
| Formate (mM) | 7.43 | 5.08 | 2.69 | 0.69 |
| pH | 6.32 | 6.48 | 0.08 | 0.32 |
| NH_3_-N (mg/100 mL) | 12.0 | 14.9 | 0.88 | 0.084 |
| Total SCFA (mM) | 94.7 | 89.9 | 5.64 | 0.70 |
| SCFA (mol/100 mol) |  |  |  |  |
| Acetate | 55.7 | 56.4 | 0.93 | 0.74 |
| Propionate | 28.5 | 26.7 | 1.69 | 0.63 |
| i-Butyrate | 0.95 | 0.75 | 0.226 | 0.69 |
| Butyrate | 11.01 | 12.20 | 0.67 | 0.41 |
| i-Valerate | 1.04 | 0.90 | 0.337 | 0.85 |
| Valerate | 1.71 | 1.93 | 0.283 | 0.73 |
| Caprionate | 1.06 | 1.12 | 0.365 | 0.94 |
| A:P | 2.08 | 2.11 | 0.156 | 0.92 |

Group 1: Treated with chloroform + phloroglucinol from day 22.

Group 2: Treated with chloroform from day 22.

**Supplementary Table 3.** Chloroform effects on DMI, CH_4_, H_2_, average daily weight gain (ADWG) and rumen fermentation parameters in steers at control period (no treatment) and day 21 of treatment (chloroform).

|  | Control | Chloroform | SEM | P-value |
| --- | --- | --- | --- | --- |
| *N* (number of animals) | 8 | 8 |  |  |
| DMI, kg | 7.3 | 6.8 | 0.203 | 0.31 |
| CH_4_ (g/kg DMI) | 22.2 | 13.2 | 0.767 | 0.001 |
| H_2_ (g/kg DMI) | 0.15 | 1.18 | 0.069 | 0.001 |
| ADWG, kg | 0.975 | 1.083 | 0.081 | 0.69 |
| Formate (mM) | 0.00 | 6.25 | 1.345 | 0.036 |
| pH | 6.48 | 6.40 | 0.063 | 0.527 |
| NH_3_-N (mg/100 mL) | 10.6 | 13.4 | 0.783 | 0.098 |
| Total SCFA (mM) | 91.8 | 92.3 | 3.28 | 0.95 |
| SCFA (mol/100 mol) |  |  |  |  |
| Acetate | 65.6 | 56.1 | 1.04 | 0.001 |
| Propionate | 21.3 | 27.6 | 1.25 | 0.015 |
| i-Butyrate | 0.87 | 0.85 | 0.121 | 0.91 |
| Butyrate | 9.96 | 11.6 | 0.516 | 0.16 |
| i-Valerate | 0.85 | 0.97 | 0.076 | 0.74 |
| Valerate | 0.96 | 1.82 | 0.139 | 0.011 |
| Caprionate | 0.44 | 1.09 | 0.202 | 0.11 |
| A:P | 3.24 | 2.10 | 0.196 | 0.007 |

- 1. **Supplementary Figures**


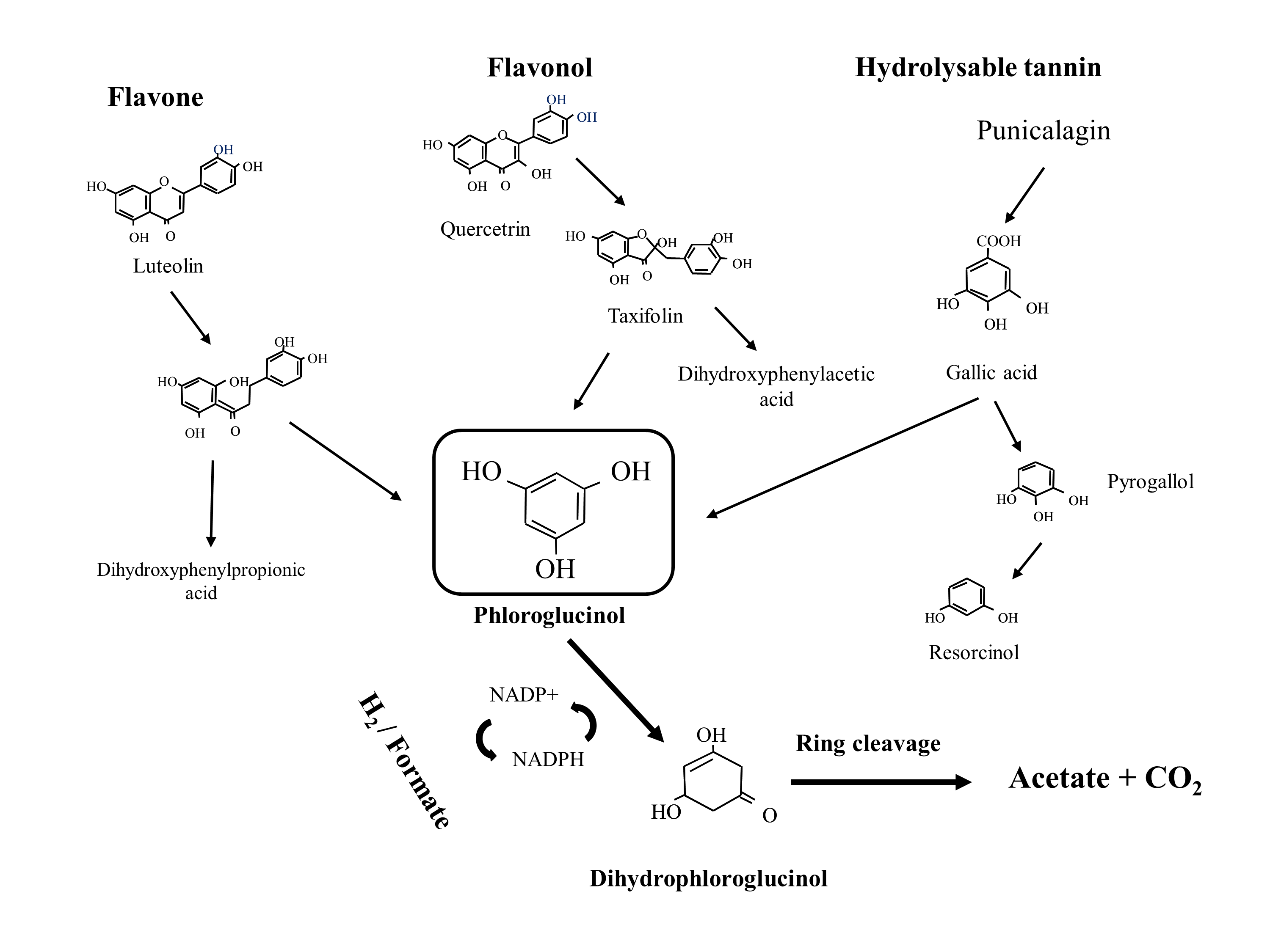


**Supplementary Figure 1.** Pathway of degradation of phloroglucinol in the rumen adapted from McSweeney et al. (2003), Conradt et al. (2016) and Tsai et al. (1976).

Conradt, D., Hermann, B., Gerhardt, S., Einsle, O., and Müller, M. (2016). Biocatalytic Properties and Structural Analysis of Phloroglucinol Reductases. *Angewandte Chemie* 128**,** 15760-15763.

McSweeney, C.S., Makkar, H.P.S. and Reed, J.D. (2003). Modification of rumen fermentation for detoxification of harmful plant compounds.  In ‘Proceedings of the 6th  International Symposium on the Nutrition of Herbivores’. eds J. Herrera-Camacho and C.A. Sandoval-Castro (Merida, Yucatan, Mexico), 239-268.

Tsai, C.G., Gates, D.M., Ingledew, W.M., and Jones, G.A. (1976). Products of Anaerobic Phloroglucinol Degradation by Coprococcus Sp Pe15. *Can J Microbiol* 22**,** 159-164.

**
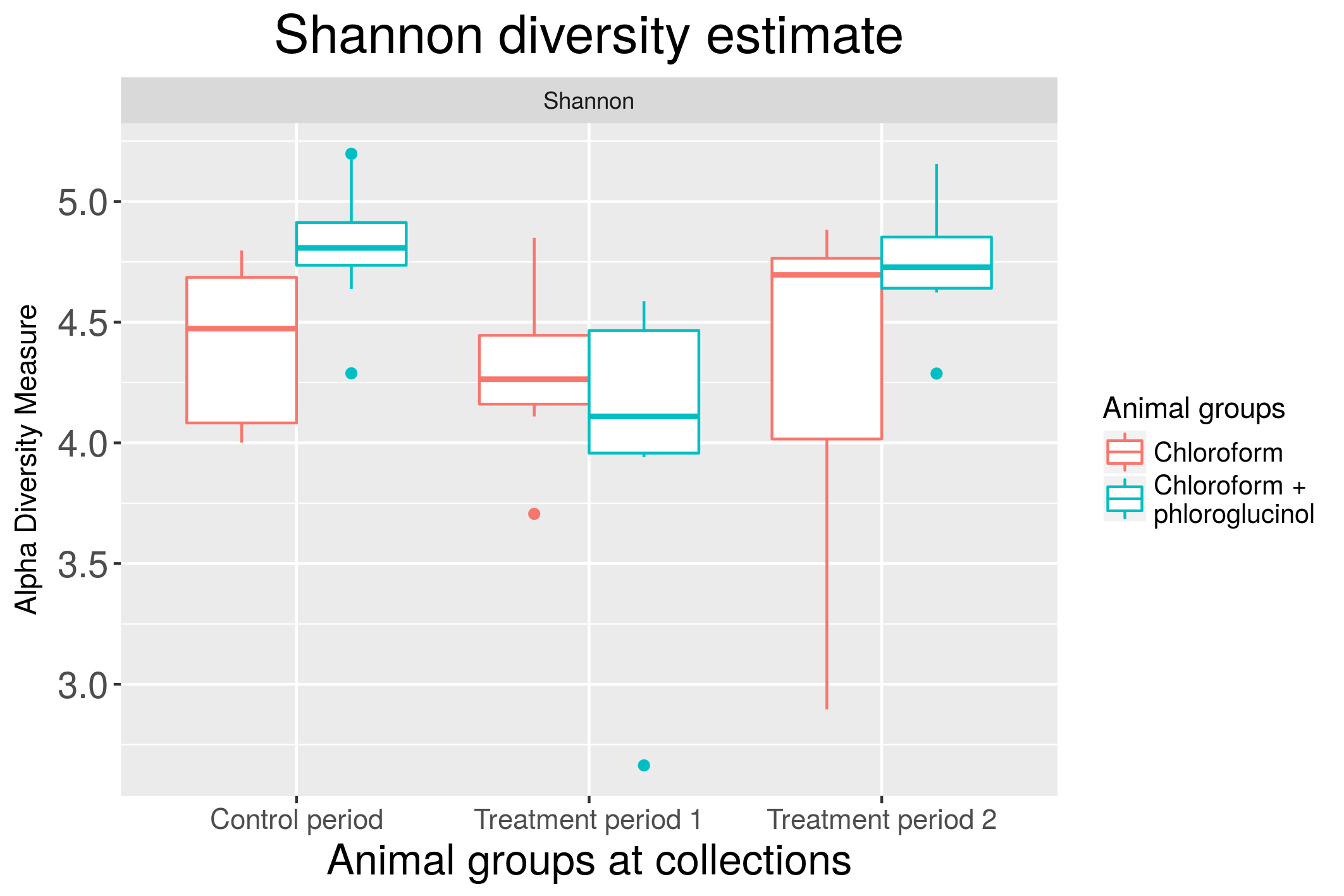
**

**
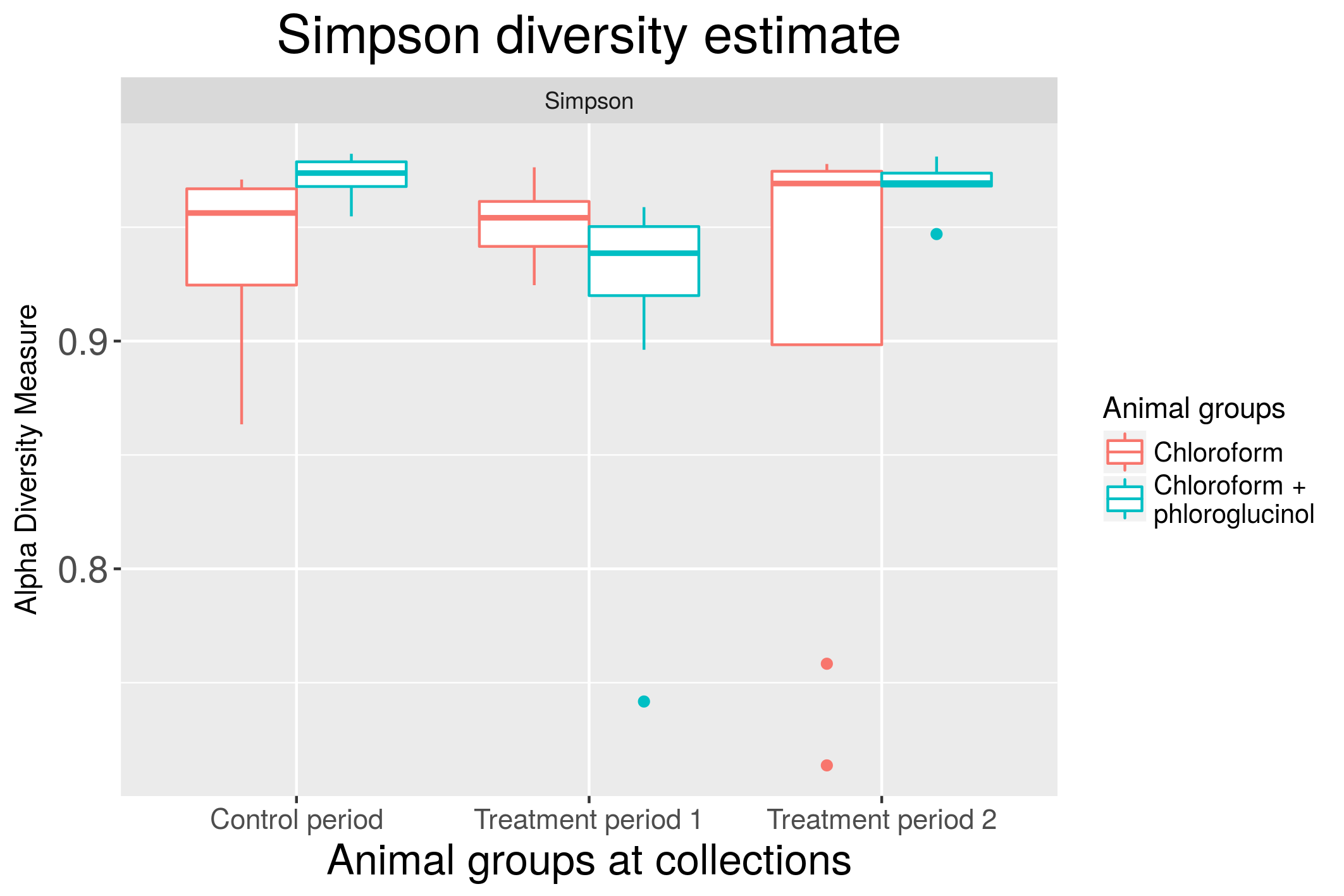
**

**Supplementary Figure 2.** Alpha diversity measures for rumen microbiomes from animals at control period (day 0), treated with chloroform (Treatment period 1, day 21) and treated with chloroform or chloroform + phloroglucinol (Treatment period 2, day 37) illustrating the Shannon diversity index (Shannon diversity estimate) and the Simpson diversity index (Simpson diversity estimate). The colors indicate the treatment that the animals received after 21 days with chloroform.


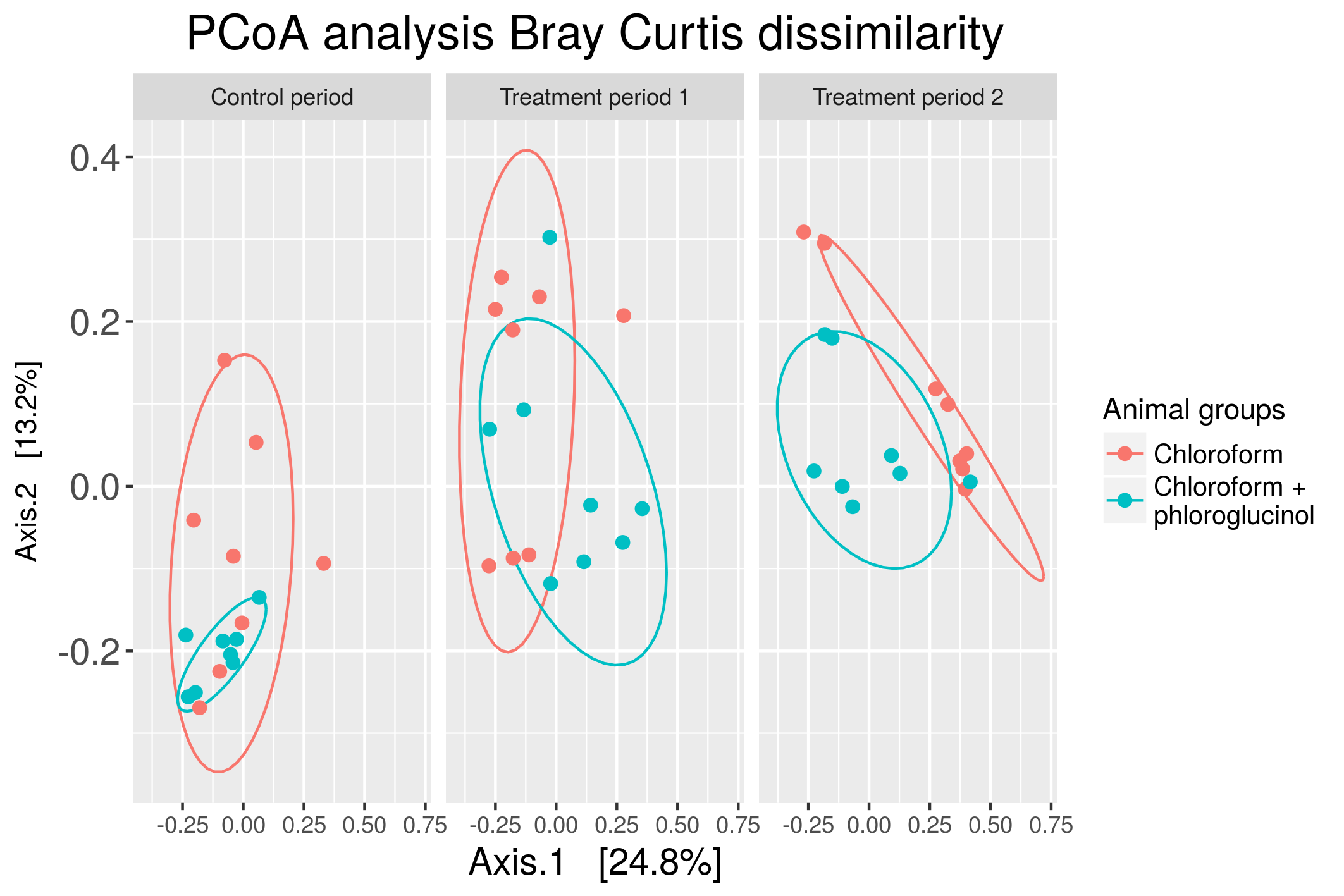


**Supplementary Figure 3.** Principle Coordinate Analysis comparing changes in microbial OTU classification based on unweighted Bray Curtis dissimilarity calculations for un-treated (control period, day 0), chloroform-treated (treatment period 1, day 21) and chloroform or chloroform + phloroglucinol treated-steers (treatment period 2, day 37). The colors indicate the treatment that the animals received after 21 days with chloroform.

**
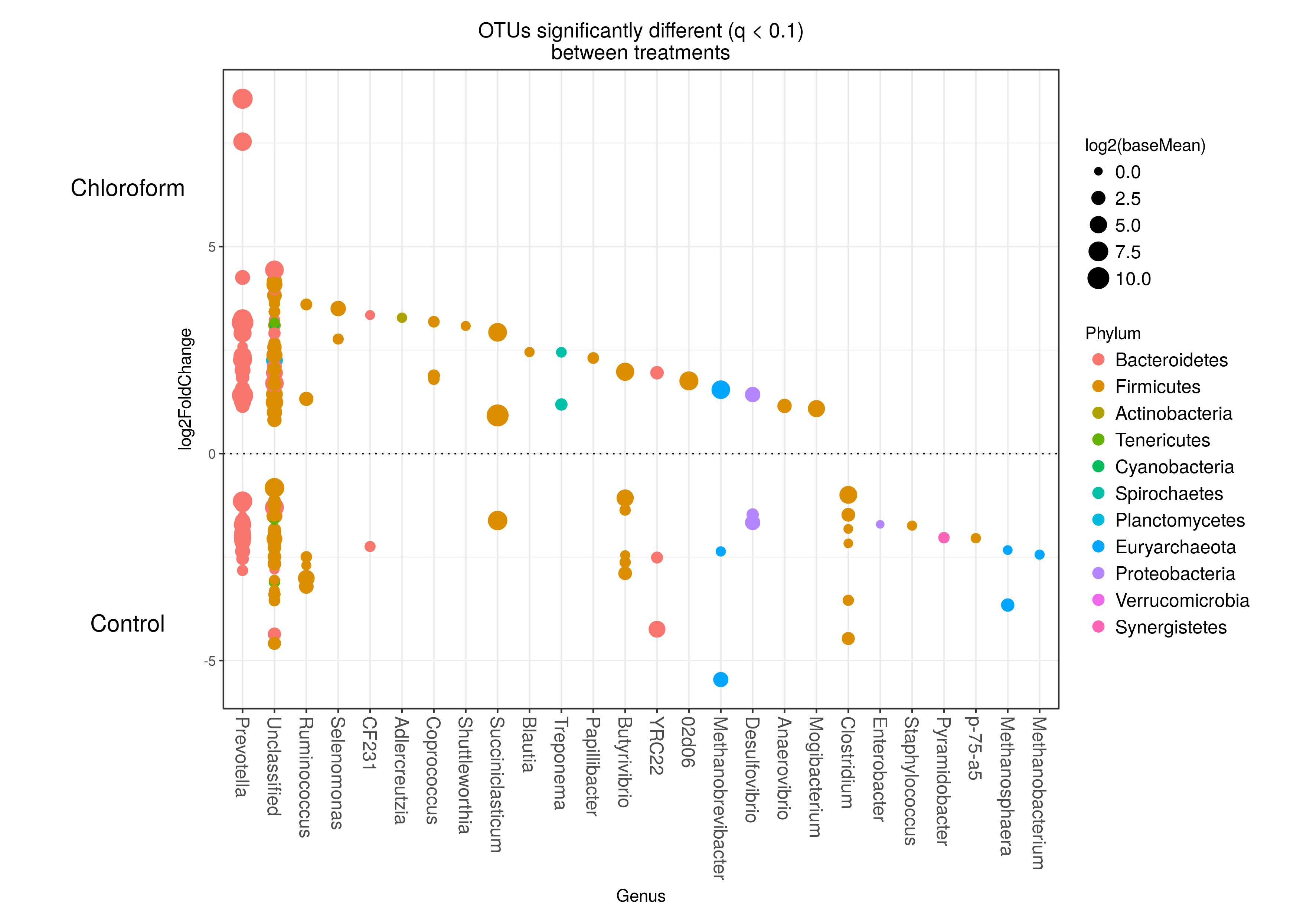
**

**Supplementary Figure 4.** OTUs significantly different (q < 0.1 FDR) between chloroform treated-animals and control period (un-treated animals). Upper axis represents OTU’s with a log2 fold positive difference for chloroform treatment relative to control while the lower y axis is the negative fold difference of the chloroform relative to control. Each point represents a single OTU colored by phylum and grouped on the x axis by taxonomic genus level, size of point reflects the log2 mean abundance of the sequence data.


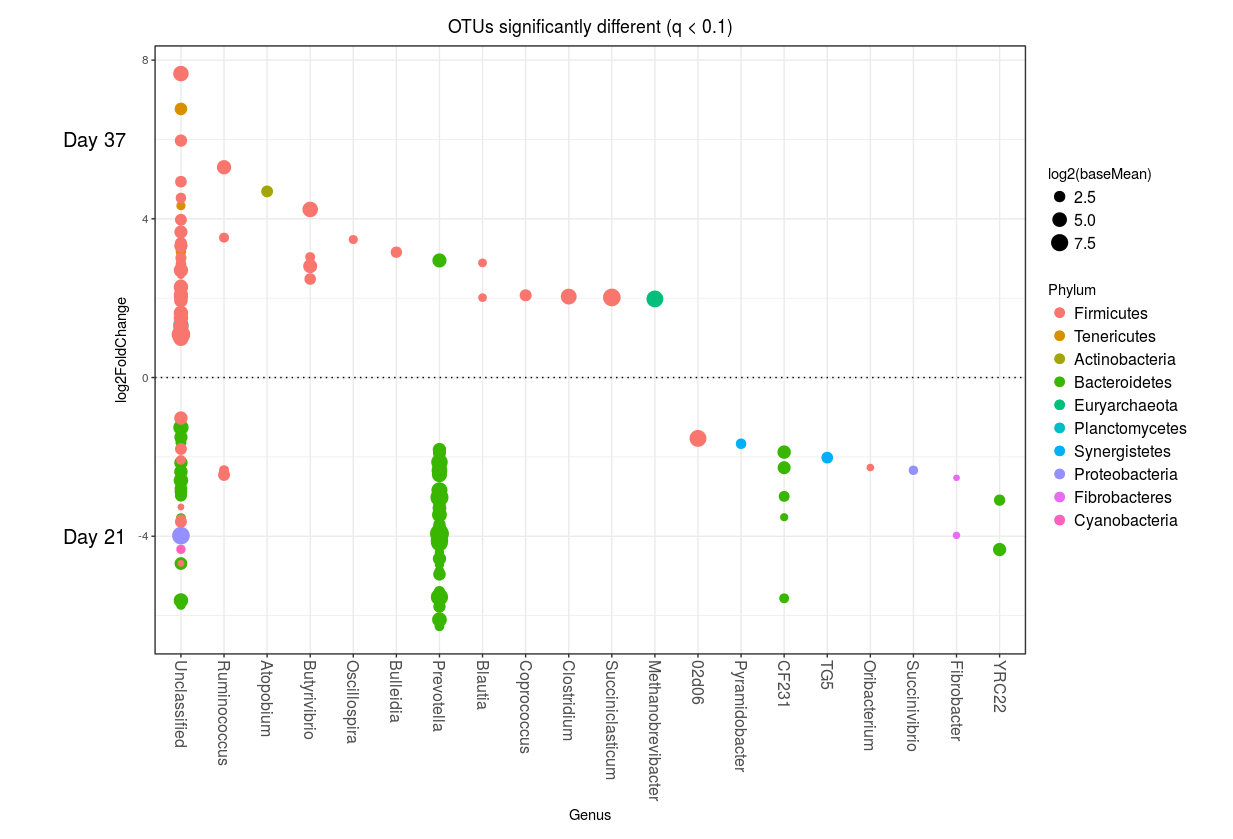


**Supplementary Figure 5.** OTUs significantly different (q < 0.1 FDR) between chloroform treated-animals at day 37 and 21. Upper axis represents OTU’s with a log2 fold positive difference for chloroform treatment at day 37 relative to chloroform at day 21, while the lower y axis is the negative fold difference of the chloroform at day 37 relative to day 21. Each point represents a single OTU colored by phylum and grouped on the x axis by taxonomic genus level, size of point reflects the log2 mean abundance of the sequence data.

**
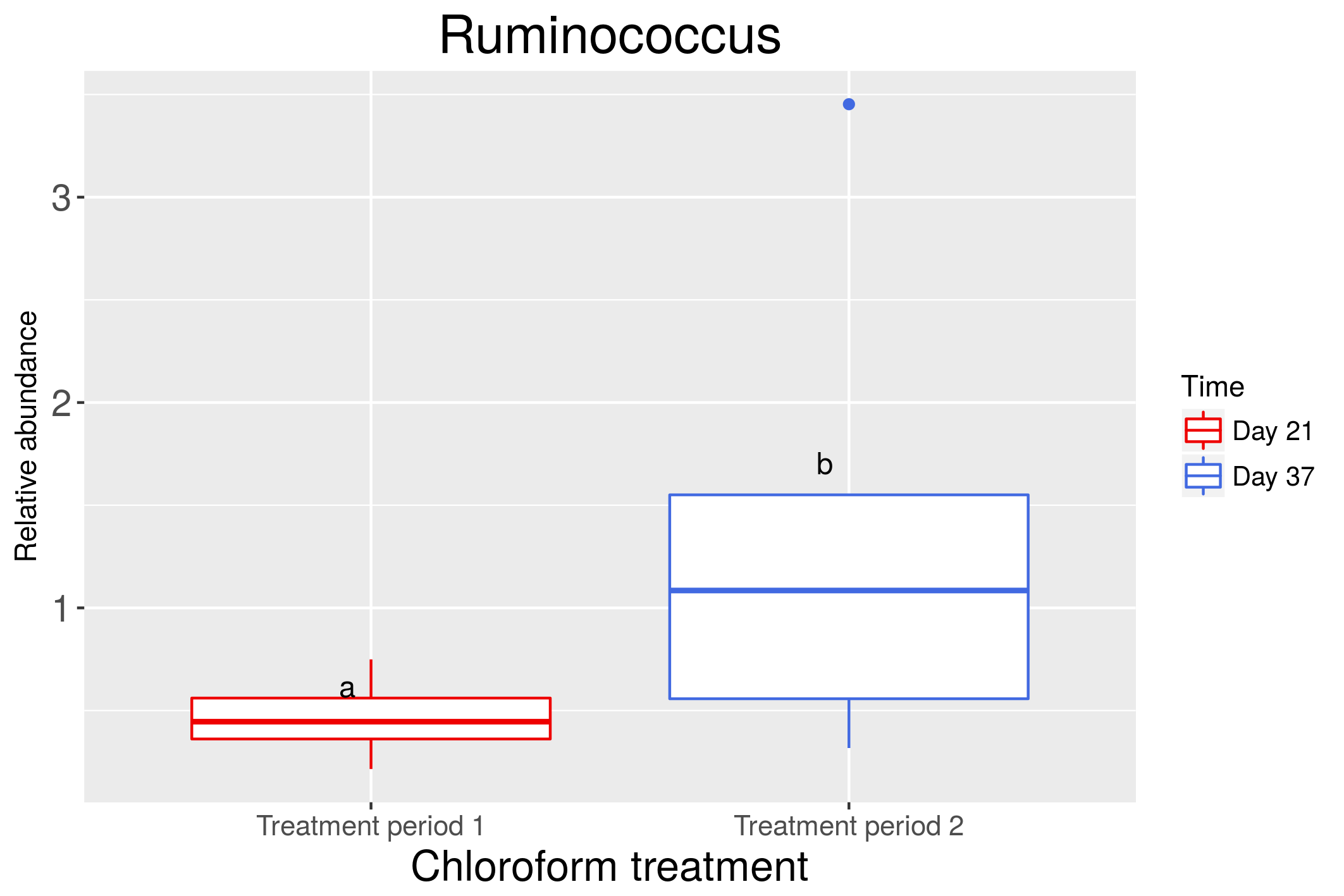
**

**Supplementary Figure 6.** Relative abundance of OTUs assigned to *Ruminococcus* genus at day 21 and 37 for chloroform treatment. ^a,b^ Letters denote significant differences between groups, bars that do not share the same letter are significantly different from each other (P < 0.05).


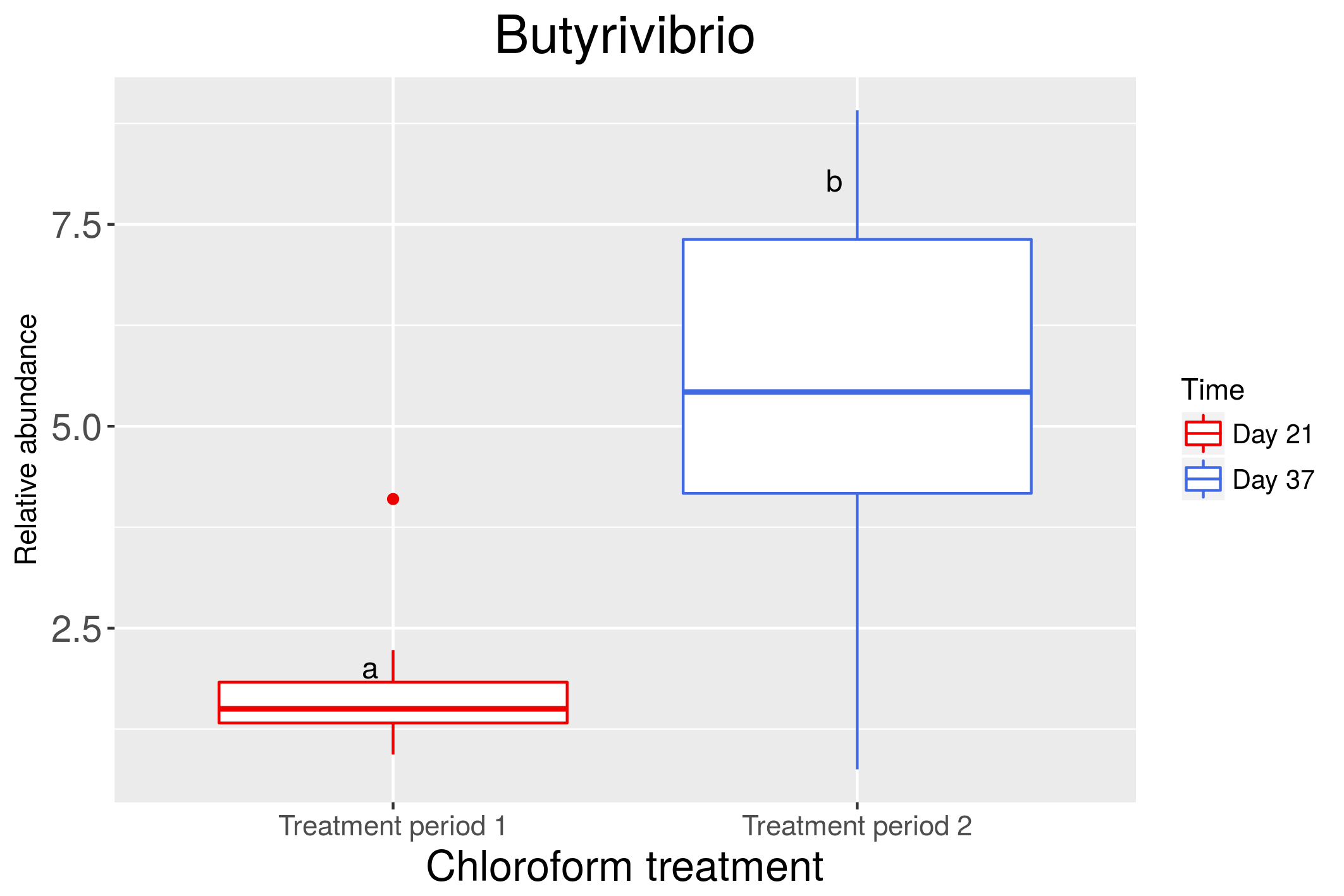


**Supplementary Figure 7.** Relative abundance of OTUs assigned to *Butyrivibrio* genus at day 21 and 37 for chloroform treatment. ^a,b^ Letters denote significant differences between groups, bars that do not share the same letter are significantly different from each other (P < 0.05).


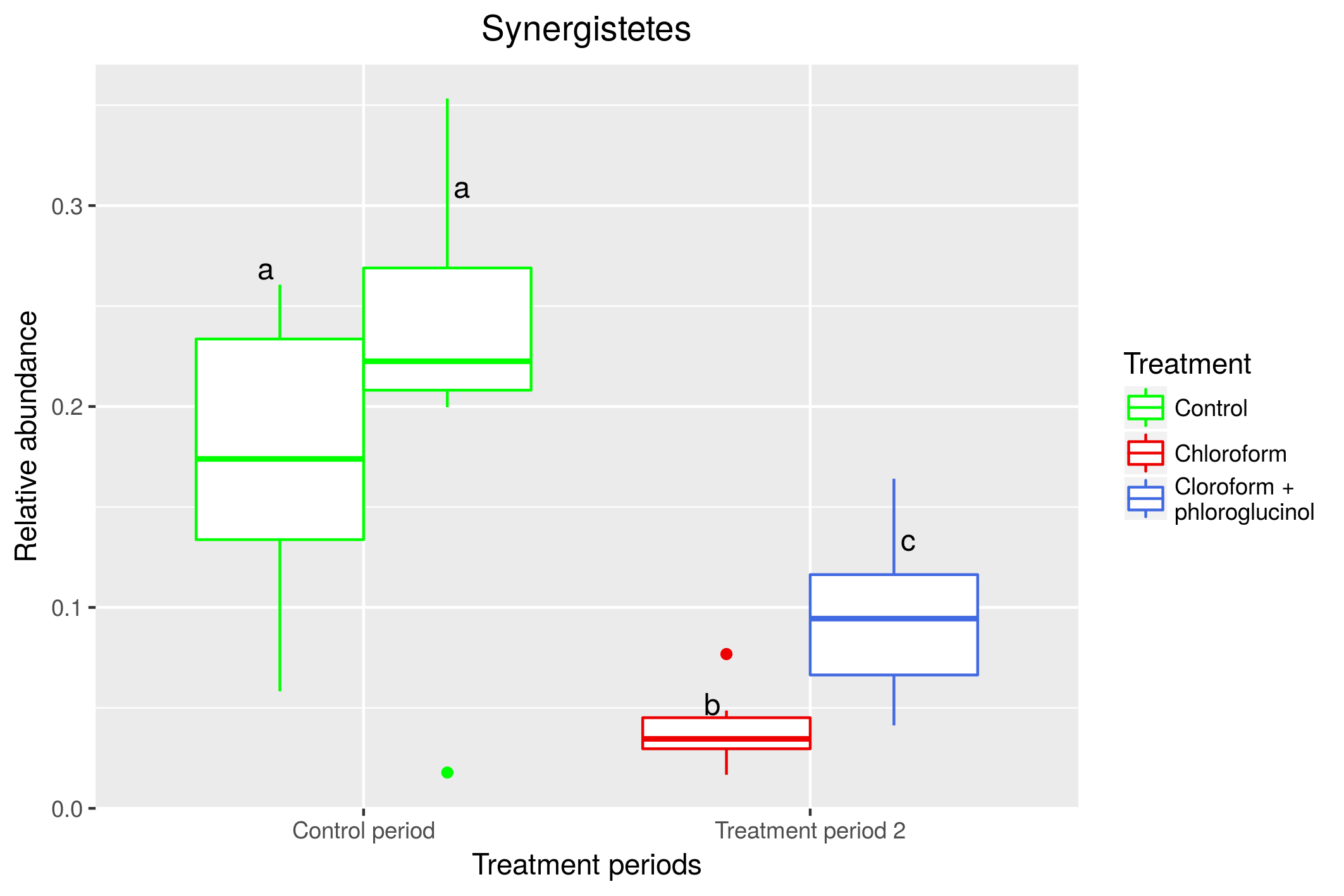


**Supplementary Figure 8.** Relative abundance of OTUs assigned to Synergistetes phylum at day 0 (Control period) and day 37 (Treatment period 2: Chloroform + phloroglucinol and chloroform-treated animals). ^a,b,c,^ Letters denote significant differences between groups, bars that do not share the same letter are significantly different from each other (P < 0.05).
